# http://Mcule.com: a public web service for drug discovery

**DOI:** 10.1186/1758-2946-4-S1-P17

**Published:** 2012-05-01

**Authors:** Robert Kiss, Mark Sandor, Ferenc A Szalai

**Affiliations:** 1 mcule.com Ltd., Budapest, H-1096, Hungary

## 

http://Mcule.com - a web service providing you a fast and cost-effective way to identify and order new drug candidates has been recently launched. The service is available for the public and it provides a comprehensive, carefully curated database of molecules immediately available for virtual screening. Several screening tools have been already implemented and more will be added on a weekly/monthly basis. Screening tools can be seamlessly integrated into a virtual screening workflow. Calculations are running on cloud machines providing a practically infinite number of CPUs and thus fast access to the screening results. Hits from the virtual screens can be ordered.

